# Updated report of *Blastocystis* spp. in a teaching hospital of Brazil: A 6-year retrospective experience

**DOI:** 10.1016/j.clinsp.2024.100543

**Published:** 2024-12-11

**Authors:** Gessica Baptista de Melo, Marcelo Alves Ferreira, Vera Lucia Pagliusi Castilho, Elenice Messias do Nascimento Gonçalves, Ronaldo Cesar Borges Gryschek, Fabiana Martins de Paula

**Affiliations:** a Laboratório de Investigação Médica (LIM 06-Laboratório de Imunopatologia da Esquistossome), Hospital das Clínicas da Faculdade de Medicina, Universidade de São Paulo, São Paulo, SP, Brazil; b Laboratório de Investigação Médica (LIM 59-Laboratório de Biologia Celular), Hospital das Clínicas da Faculdade de Medicina, Universidade de São Paulo, São Paulo, SP, Brazil; c Seção de Parasitologia, Divisão do Laboratório Central, Hospital das Clínicas, Faculdade de Medicina, Universidade de São Paulo, São Paulo, SP, Brazil

*Blastocystis* spp. is an intestinal enteric protist member of the phylum Stramenopiles, with a high occurrence around the world that can vary from region to region, according to hygienic and sanitary conditions.^1^ In recent years, there has been a growing number of studies on the distribution of Subtypes (STs) and their association with the microbiota, as well as the possibility of causing disease.^2^, ^3^, ^4^ Even so, many issues remain controversial, since the reported results differ widely according to the selection and sampling strategy, diagnostic method and subtyping.^3^

In a recent literature review, the occurrence of *Blastocystis* spp. in Brazil ranges from 0.5% to 86.6%, considering diagnosis by parasitological techniques.^5^ Several studies have been carried out in the state of São Paulo, but none have addressed the frequency of *Blastocystis* spp. in samples from patients at the Hospital das Clinicas da Faculdade de Medicina da Universidade de São Paulo (HC-FMUSP) on a large scale over a long period. Therefore, the aim of this study was to evaluate the positivity of *Blastocystis* spp. in routine parasitological tests carried out by the Parasitology Section of the Central Laboratory Division of HC-FMUSP between 2016 and 2021.

This is a retrospective descriptive analysis study carried out in collaboration with the Parasitology Section of the Central Laboratory Division (SPD-DLC, HC/FMUSP). A database was created with information on the results of stool parasitological tests recorded between January 1, 2016, and December 31, 2021. Only the first result of individuals with several parasitological results was included. The techniques used at SPD-DLC to issue stool parasitology results are the Lutz, Faust, permanent Trichrome, and Leishman staining methods.^6^

Between 2016 and 2021, SPD-DLC analyzed 15,438 stool samples from 8561 patients. Of this total, 500 randomized medical records were included (with test power equal to 0.8 in the analysis). Individual data such as gender, age, place of residence, referral service, and parasitological result were anonymized and recorded in this database. Categorical variables were compared using Pearson's χ2 test. A Probability (P) of less than or equal to 0.05 was considered statistically significant. The test power achieved with the sample used was 0.799 and the effect size (ω) was 0.16. All statistical analyses were carried out on the *R* platform for statistical programming (version 4.1.2).

The distribution of the frequency of parasitological results over the period studied was not homogeneous (p = 0.02394), with an increase in cases between 2017 and 2020 (Table 1). Of the total number of individuals, 285 (57.0%) and 215 (43.0%) were female and male, respectively, with ages ranging from under one year to 92 years (39.6±22.2).

**Table 1:** Demographic data of the 500 medical records included in the study.

Characteristics		Total	Positive	Negative	p-value
Gender, n (%)	Female	285 (57.0)	203 (70.2)	82 (28.8)	0.3622
	Male	215 (43.0)	145 (67.4%)	70 (32.6)	
Age (average ± DP)	< 1 to 92-years	500	41.2 (±21.0)	35.9 (±24.4)	0.0316
Location, n (%)	City SP	278 (55.6)	76 (27.3)	202 (72.7)	0.1862
	Greater SP	158 (31.6)	107 (67.3)	51 (32.3)	
	Other locations	64 (12.8)	39 (60.9)	25 (39.1)	
Requesting department, n (%)	DHAS	2 (0.4)	2 (100)	0	
	HLS	4 (0.8)	4 (100)	0	
	ICHC	313 (62.6)	229 (73.2)	84 (26.8)	
	ICR	102 (20.4)	50 (49.0)	52 (51.0)	
	ICSP	23 (4.6)	10 (43.5)	13 (56.5)	1.3992e-07
	IMREA	1 (0.2)	1 (100)	0	
	INCOR	15 (3.0)	15 (100)	0	
	IOT	6 (1.2)	4 (66.7)	2 (33.3)	
	IPQ	8 (1.6)	8 (100)	0	
	PA	2 (0.4)	2 (100)	0 (0)	
	CACEC	24 (4.8)	23 (95.8)	1 (4.2)	
Year, n (%)	2016	70 (14.0)	41 (58.6)	29 (41.4)	0.02394
	2017	96 (19.2)	70 (72.9)	26 (27.1)	
	2018	101 (20.2)	80 (79.2)	21 (20.8)	
	2019	100 (20.0)	72 (72.0)	28 (28.0)	
	2020	64 (12.8)	44 (68.7)	20 (31.3)	
	2021	68 (13.6)	40 (58.8)	28 (41.2)	

DHAS, Departamento Hospital Auxiliar de Suzano; HLS, Hospital Local Sapopemba; ICHC, Instituto Central Hospital das Clínicas; ICR, Instituto da Criança e do Adolescente; ICSP, Instituto do Câncer do estado de São Paulo; IMREA, Instituto de Medicina Física e Reabilitação; INCOR, Instituto de Coração; IOT, Instituto de Ortopedia e Traumatologia; IPQ, Instituto de Psiquiatria; PA, Pronto Atendimento; CACEC, Centro de Atendimento ao Colaborador.

The results of the parasitological tests revealed that *Blastocystis* spp. was found in 69.6% (348) of the stool samples. There was no statistically significant difference between gender and *Blastocystis* positivity (p = 0.3622). With regard to age, the average among the positives was 41.2 ± 21.0, and among the negatives 35.9 ± 24.4 (p = 0.0316). In addition to *Blastocystis* spp. Other species of intestinal parasites were observed, such as *Entamoeba histolytica/dispar, Giardia intestinalis* and *Crystosporidium* sp.

The factors involved and the consequence of the high frequency of *Blastocystis* spp. in the world remain to be clarified. The results found here confirm the high positivity of *Blastocystis* spp. from other studies carried out in the state of São Paulo.^7^^,^^8^ In contrast, surveys carried out in different regions of the state of São Paulo indicate a lower average positivity, using different techniques for parasitological diagnosis from those employed in the SPD-DLC.^9^^,^^10^

Regarding the distribution of positive cases and regions in the city of São Paulo, the majority came from the municipality of São Paulo, with 278 (55.6%), followed by the greater São Paulo area with 158 (31.6%) and others with 64 (12.8%) (p = 0.1862). Similarly, the positivity between regions was higher in the West, followed by the Center and East (p = 0.1558). It was expected that there would be greater positivity in those regions with a lower human development index.

It is interesting to note that there was a high proportion of positive cases in organ transplant services (28.4%), gastroenterology (10.9%), dermatology (11.5%) and infectious diseases (7.7%), which may be associated with a greater request for parasitological tests in these services, and not with the frequency of *Blastocystis* spp. infection (Fig. 1). Reinforcing these findings, our group evaluating patients with urticaria^11^ and transplant candidates treated at HC-FMUSP^12^ pointed to high positivity for *Blastocystis* spp. Additionally, the literature has highlighted the high positivity of *Blastocystis* in immunocompromised individuals, as well as the possibility of infection by this organism being associated with clinical manifestations such as diarrhea, irritable bowel syndrome, and chronic urticaria.^2^^,^^4^ Although this association is the subject of discussion.

**Fig. 1:**
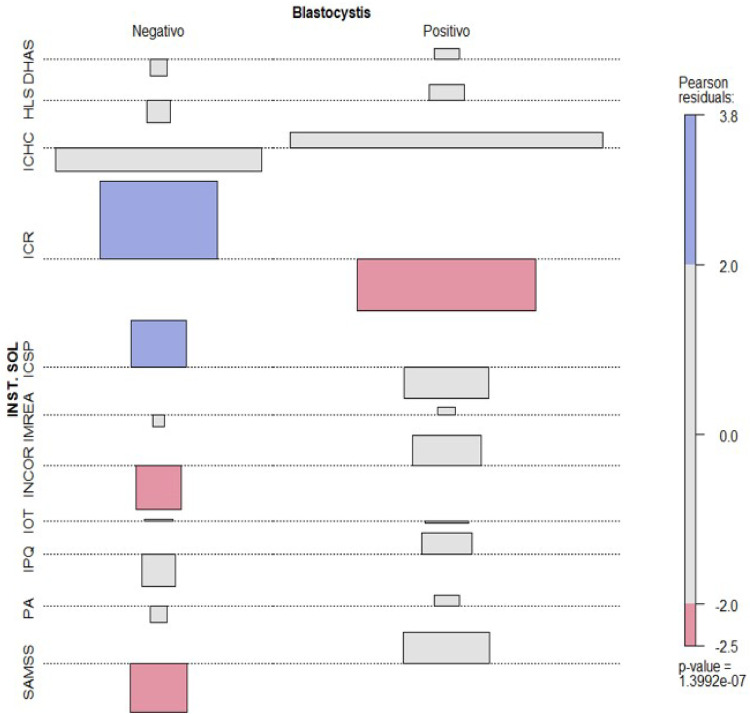
Origin of stool samples analyzed between 2016 and 2021 at the Parasitology. Section of the Instituto Central do Hospital das Clínicas da Universidade de São Paulo, in relation to the requesting service DHAS (Departamento Hospital Auxiliar de Suzano), HLS (Hospital Local Sapopemba), ICHC (Instituto Central Hospital das Clínicas), ICR (Instituto da Criança e do Adolescente), ICSP (Instituto do Câncer do Estado de São Paulo), IMREA (Instituto de Medicina Física e Reabilitação), INCOR (Instituto de Coração), IOT (Instituto de Ortopedia e Traumatologia), IPQ (Instituto de Psiquiatria), PA (Pronto Atendimento), CACEC (Centro de Atendimento ao Colaborador).

The limitations of this study are mainly related to the lack of clinical and epidemiological data, which made it impossible to establish an association between *Blastocystis* spp. and symptoms or environmental factors. Overall, the data indicate intense positivity of this organism in the city of São Paulo, which may suggest that environmental conditions may be favoring its circulation, and reinforces the need for a better understanding of this finding in the context of public health.

## Statement of ethics

The Research Ethics Committee of the Department of Infectious and Parasitic Diseases of the Faculty of Medicine of the University of São Paulo and the Research Ethics Committee (CAPPesq) of HC-FMUSP approved this study (n° 3.325.341).

## Authors’ contributions

Melo GB and Paula FM contributed to the conception and design of the study. Castilho VLP and Gonçalves EMN performed the clinical results search. Ferreira MA carried out the statistical analysis. Melo GB and Paula FM wrote the manuscript. Melo GB, Paula FM, Castilho VLP, Gonçalves EMN and Gryschek RCB reviewed and approved the final version of the manuscript.

## Funding

No funding was received.

## Declaration of competing interest

The authors declare no conflicts of interest.
